# Meal Replacement Provides Additional Benefits for Weight and Visceral Fat Reduction in Prediabetic Individuals: A Randomized Clinical Trial

**DOI:** 10.1155/jnme/7109903

**Published:** 2026-08-24

**Authors:** Ye Zhou, Lunan Jin, Pingping Zhang, Yanni Li, Yong Jin, Miao Xu, Qifa Song, Li Li

**Affiliations:** ^1^ Department of Endocrinology and Metabolism, The First Affiliated Hospital of Ningbo University, Ningbo, Zhejiang, China, nbu.edu.cn; ^2^ Hangzhou Qingwu Health Management Co., Hangzhou, Zhejiang, China; ^3^ Ningbo Center for Healthy Lifestyle Research, The First Affiliated Hospital of Ningbo University, Ningbo, Zhejiang, China, nbu.edu.cn; ^4^ School of Medicine and Dentistry, Griffith University, Gold Coast Q4222, Queensland, Australia, health.qld.gov.au; ^5^ Department of Infection, Ningbo Yinzhou No.2 Hospital, Ningbo, Zhejiang, China; ^6^ Medical Data Research Center, The First Affiliated Hospital of Ningbo University, Ningbo, China, nbu.edu.cn

**Keywords:** lifestyle intervention, meal replacement, overweight and obesity, prediabetes

## Abstract

**Background:**

Meal replacements (MRs) have been shown to improve cardiometabolic risk factors; however, their effects on visceral fat and metabolic characteristics in individuals with prediabetes remain insufficiently investigated. This study was aimed to demonstrate the effectiveness of MR combined with lifestyle intervention in improving weight, visceral fat area (VFA), blood pressure, and biochemical indicators in participants with prediabetes comorbid with overweight/obesity.

**Materials and Methods:**

According to the inclusion and exclusion criteria, 100 prediabetic participants (25 kg/m^2^ ≤ body mass index [BMI] ≤ 35 kg/m^2^) were recruited and randomly divided into the lifestyle intervention group (LSI group; received guidance on lifestyle interventions) and the lifestyle intervention combined with MR group (LSI + MR group; received a nutrient‐rich and low‐carbohydrate MR to replace the staple food of two meals or replace one regular meal while receiving lifestyle interventions). Forty‐two participants in the LSI group and 45 in the LSI + MR group completed the trial. Anthropometric indexes were collected and biochemical indexes were tested at baseline and 12 weeks post‐intervention.

**Results:**

Compared with the LSI group, the LSI + MR group showed greater percentage reductions in weight (−5.3% ± 5.5% vs. −8.3% ± 4.5%; *p* < 0.01) and VFA (−11.9% ± 21.0% vs. −22.7% ± 23.8%; *p* < 0.05). And there were significant time × group interaction effects for weight (*F* = 5.296, *p* = 0.023), BMI (*F* = 5.124, *p* = 0.025), and VFA (*F* = 9.177, *p* = 0.003). For blood pressure, postprandial glucose, insulin resistance, liver enzymes, and lipids, there were only time effects (*p* < 0.05) but no time × group interaction effects (*p* > 0.05). Subgroup analyses suggested that MR‐related benefits varied by baseline VFA, with greater improvement in 2‐h postprandial plasma glucose among participants with VFA < 100 cm^2^ and greater reductions in weight, triglyceride, and total cholesterol among those with VFA ≥ 100 cm^2^.

**Conclusion:**

Lifestyle intervention combined with MR resulted in greater reductions in body weight and VFA than lifestyle intervention alone in individuals with prediabetes and overweight/obesity. The additional metabolic benefits of MR appeared to vary according to baseline visceral adiposity.

**Trial Registration:** ClinicalTrials.gov identifier: NCT05273840

## 1. Introduction

Prediabetes is defined as a fasting plasma glucose (FPG) level between 100 and 125 mg/dL, a 2‐h postload glucose level of 140–199 mg/dL following a 75‐g oral glucose tolerance test, or a glycated hemoglobin (HbA1c) level ranging from 5.7% to 6.4% [1]. Approximately 5%–10% of patients with prediabetes progress to diabetes each year [2]. Studies have shown that prediabetes is strongly associated with all‐cause mortality, cardiovascular disease (CVD), microangiopathy, retinopathy, tumors, dementia, mental disorders, and diabetic foot ulcers [3]. An observational cohort study demonstrated that a reversion to normoglycemia from prediabetes resulted in a lower risk of CVD events and death [4]. More recently, post hoc analyses from two landmark diabetes prevention trials, the Diabetes Prevention Program Outcome Study (DPPOS) and the DaQing Diabetes Prevention Outcome Study (DaQingDPOS), further showed that remission of prediabetes was associated with a substantially lower long‐term risk of cardiovascular death or hospitalization for heart failure [5]. Therefore, the effective management of prediabetes, particularly strategies that promote restoration of normal glucose regulation, is important not only for preventing diabetes but also for reducing long‐term cardiovascular risk.

Currently, intervention strategies for prediabetes focus on pharmacological interventions, health education, and lifestyle interventions or a combination of all of these interventions [1]. It has been found people with prediabetes who were educated about prediabetes and diabetes self‐management can effectively improve their behaviors and lifestyles [6] and delay or prevent the development of diabetes [7, 8]. For example, a 6‐year lifestyle intervention reduced the cumulative incidence of progression to diabetes over the next 30 years by 39% in people with impaired glucose tolerance [9]. Thus, lifestyle intervention remains a cornerstone of prediabetes management.

Lifestyle interventions typically include nutritional dietary modification and physical activity. One feasible dietary strategy is the use of meal replacements (MRs), which provide controlled calories and nutrients to facilitate weight control. A recent meta‐analysis has shown MRs significantly improve key cardiometabolic markers in individuals with prediabetes and metabolic syndrome [10]. However, the biological heterogeneity of prediabetes remains largely overlooked [11]. Emerging evidence has identified distinct high‐risk prediabetes subtypes characterized by increased visceral and hepatic fat accumulation, which may drive progression toward metabolic complications even before a diabetes diagnosis [11].

Although visceral adipose tissue is recognized as a critical contributor to the development of type 2 diabetes (T2DM) [12], and its reduction is independently linked to significant improvements in cardiometabolic markers like HbA1c and lipid profiles [13], visceral fat has seldom been evaluated as a primary outcome in MR‐based interventions. Therefore, the present randomized clinical trial aimed to examine whether adding MR to lifestyle intervention could further improve visceral fat area (VFA) and metabolic characteristics in individuals with prediabetes and overweight/obesity.

## 2. Materials and Methods

This study is a prospective, single‐center, randomized clinical trial. Approval of protocol was obtained from the ethics committee of the Ningbo First Hospital (Approval No. 2021‐R037, approved June 1, 2021). The study was conducted in accordance with the ethical standards in the Declaration of Helsinki. All participants were fully informed of the study protocol and signed an informed consent form prior to their participation in the study.

### 2.1. Participants and Randomization

Participants with prediabetes (FPG 6.1–6.9 mmol/L or 2‐h postprandial glucose (2hPG) 7.8–11.0 mmol/L or HbA1c 5.7%–6.4%), overweight or obesity (body mass index [BMI]: 25–35 kg/m^2^), and aged 18–70 years were included in this study.

Participants were excluded if they met any of the following criteria: diagnosed diabetes mellitus; weight change of more than 10% in the last 3 months; pregnant or lactating women; having diseases that are unsuitable for participation in this study, such as severe liver dysfunction, kidney dysfunction, severe mental illness, and others; allergic to the ingredients of the nutritional supplement; or participation in other clinical trials in the last 3 months.

The study used a simple randomized design with seeds and random numbers generated by STATA software (StataCorp LP, College Station, TX) to randomly group the 100 numbers into two groups: lifestyle intervention (LSI group) and lifestyle intervention combined with MR (LSI + MR group). Grouping information was filled into opaque, consecutively labeled envelopes, which were centrally managed by a single researcher, and participants were enrolled sequentially according to the grouping information in the envelopes [14]. Both participants and healthcare providers were aware of group allocation.

### 2.2. Sample Size

The sample size was estimated based on expected differences in absolute change in body weight between groups. Based on a previous study, in which the LSI group experienced a weight loss of −1.9 kg with 95% CI [−3.5; −0.4] and the MR group lost −5.6 kg with 95% CI [−6.6; −4.5] over 12 weeks [15], we anticipated a between‐group difference of approximately 3.5 kg, with an estimated standard deviation (SD) of 5.0 kg.

To detect this difference with a power of 80% and a two‐sided significance level of 0.05, a minimum of 33 participants per group was required, calculated using a two‐sample *t*‐test. To improve statistical power and account for an anticipated dropout rate of approximately 20%, we aimed to enroll a total of 100 participants (50 per group).

### 2.3. MR Products

The MR product (iVital Control, Solid Bev. Choc.; Fresenius Kabi Deutschland GmbH, Bad Homburg, Germany) in this study was nutrient‐rich and low‐carbohydrate. Its main ingredients included 18% isolated whey protein powder and 11% medium‐chain triglycerides, with several low‐concentration additives of modified starch and fructose, multivitamins, and minerals. Its energy density was 1.06 kcal/mL. This MR was packed in a package of 46 g powder that had 212 kcal (885 kJ) energy, 19.2 g carbohydrate, 9.2 g protein, 10.2 g fat (*C*: *P*: *F* = 37: 18: 42, % kcal), and 3.5 g fiber. One package was added to 160 mL of warm water and then stirred until completely dissolved before drinking.

The participants in the LSI + MR group were required to take either of the following ways to consume MR: 2 packets once a day to replace any one regular meal or 1 packet twice a day to replace the staple food of two regular meals. This study was conducted in eastern China, where rice is commonly consumed as the primary staple food. Compliance with the MR regimen was based on participant self‐administration following standardized verbal and written instructions at enrollment.

### 2.4. Lifestyle Intervention

Both groups received the same standardized lifestyle intervention program consisting of dietary counseling, exercise guidance, and behavioral education. To minimize performance bias, the intervention framework, counseling content, visit schedule, follow‐up procedures, and feedback principles were identical between the two groups and were delivered by the same team of trained dietitians. The only difference between groups was the inclusion of MRs in the LSI + MR group.

Participants in both groups received face‐to‐face counseling at each visit. Individualized lifestyle prescriptions were developed by dietitians based on baseline body weight and lifestyle diaries, following a standardized framework. All participants were enrolled in designated WeChat groups for remote guidance. They were required to upload photos of their meals at least once daily and of their physical activity at least three times per week. Based on these records, dietitians provided consistent feedback and made adjusted recommendations according to predefined principles.

As to exercise, each participant was instructed to complete moderate aerobic exercise at least four times a week. The duration of each session was at least 40 min. The exercise mainly included brisk walking, jogging, swimming, various ball games, calisthenics, and so on. For dietary intervention, a standardized low‐energy diet was prescribed in accordance with the Chinese Consensus on Prediabetes Intervention. This diet consisted of approximately 45%–60% energy from carbohydrate, 25%–35% from fat, and 15%–20% from protein [16]. The total daily calorie requirement was calculated as follows: total calorie (kcal) = actual body weight (kg) × calorie requirement per kilogram of body weight (kcal/kg) × 0.7. The amount of calorie requirement per kilogram of body weight was based on different occupations (labor intensity): 20–25, 30, and 35 kcal/kg for light, medium, and heavy work, respectively.

### 2.5. Follow‐Up and Metabolic Indicators Collection

The participants were followed up for 3 months with 3 scheduled visits. Anthropometrical (weight, height, waist circumference, and hip circumference), clinical (blood pressure, VFA, and subcutaneous fat area [SFA]), and biochemical indicators were measured at baseline and the last visit. Anthropometric measurements were performed by well‐trained endocrinology nurses. Body weight was measured in light clothing to the nearest 0.1 kg and height to the nearest 0.1 cm. Waist circumference was measured at the minimum abdominal girth (midway between the rib cage and the iliac crest). Hip circumference was measured at the widest point over the buttocks. Blood pressure was measured using an electronic sphygmomanometer on both arms after a five‐minute rest in a sitting position, and the average blood pressure was obtained on two assessments. VFA and SFA were measured at the level of the umbilicus by bioelectrical impedance analysis (DUALSCAN HDS‐2000, Omron Healthcare Kyoto, Japan).

Venous blood was collected after an overnight fast of at least 10 h, and biochemical indicators included FPG, fasting insulin (FINS), HbA1c, liver function, uric acid, and blood lipids. Then, the 75 g oral glucose tolerance test was performed in all subjects to assess the 2hPG and 2‐h postprandial insulin (2hINS) concentrations. FPG and 2hPG were assayed by the glucose oxidase method, FINS and 2hINS were determined by chemiluminescence immunoassay. Lipid profiles were analyzed by enzymatic procedures using an autoanalyzer (Hitachi 747; Hitachi, Tokyo, Japan). HbA1c was determined by high‐pressure liquid chromatography. UA was examined by enzymatic spectrophotometry.

### 2.6. Outcomes

The primary outcome of this study was the percentage change in body weight from baseline to 12 weeks. Secondary outcomes included changes in anthropometric measures (e.g., BMI, waist circumference, VFA), blood pressure, glycemic parameters, liver function, uric acid, and lipid profiles.

### 2.7. Statistical Analysis

Continuous variables were tested for normality using the Kolmogorov–Smirnov test. Normal and skewed distributed variables were presented as mean ± SD and median (interquartile range), respectively. Changes between the anthropometric values within the groups were compared using the paired‐sample *t*‐tests. Between‐group comparisons of change values were performed using independent samples *t*‐tests for continuous variables. Univariate general linear models were used to analyze the effects of LSI + MR, including time effects, group effects, and the interaction effects of time × group. The primary analysis was conducted among participants who completed the 12‐week follow‐up. For variables with missing values among these participants, multiple imputation was used under the assumption that data were missing at random.

An exploratory subgroup analysis was conducted according to baseline VFA to examine whether the effects of MR differed by visceral adiposity status. Participants were stratified into two subgroups: VFA < 100 cm^2^ and VFA ≥ 100 cm^2^. Within each subgroup, changes from baseline to 12 weeks were calculated for anthropometric and metabolic indicators, and between‐group comparisons of these change values were performed between the LSI and LSI + MR groups.

Results were considered statistically significant at a two‐tailed level of 0.05. No formal adjustment for multiple comparisons was applied for secondary outcomes or exploratory subgroup analyses; therefore, these findings should be interpreted with caution. Statistical analyses were performed with IBM SPSS Statistics Version 25.0.

## 3. Results

### 3.1. Study Flow and Baseline Characteristics

**FIGURE 1:**
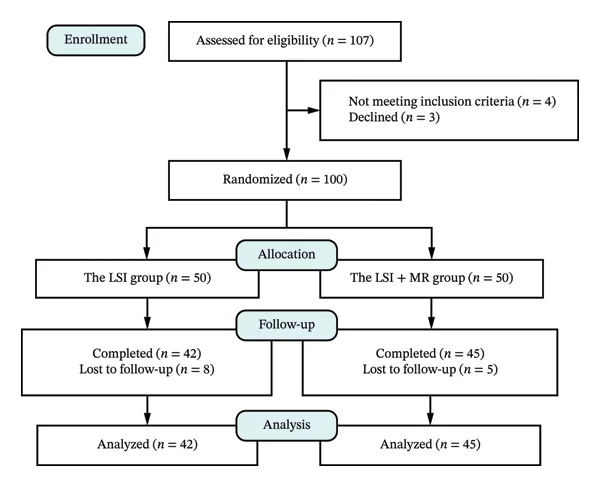
Flowchart of the study.

Following the application of inclusion and exclusion criteria, 100 out of 107 individuals with prediabetes comorbid with overweight/obesity were enrolled and randomized in a 1:1 ratio to either the lifestyle intervention (LSI) group (*n* = 50) or the lifestyle intervention plus meal replacement (LSI + MR) group (*n* = 50). During the follow‐up period, eight participants from the LSI group and five from the LSI + MR group discontinued the intervention due to noncompliance, scheduling conflicts, or other unspecified reasons. Ultimately, 87 participants (87%) completed the study, while 13 (13%) were lost to follow‐up (Figure 1).

The baseline characteristics of the participants are shown in Table 1. For the LSI group and the LSI + MR group, the mean age of the subjects was 33.2 ± 6.7 and 34.7 ± 9.0 years, and the body weight was 81.4 ± 12.6 kg and 82.2 ± 12.7 kg, respectively. There were no significant differences in baseline characteristics between the two groups (Table 1).

**TABLE 1:** Baseline characteristics of LSI group and LSI + MR group.

	LSI group (*n* = 50)	LSI + MR group (*n* = 50)	*p*
Gender (% male)	19 (38%)	23 (46%)	0.544
Age (years)	33.2 ± 6.7	34.7 ± 9.0	0.348
Height (cm)	163.7 ± 7.6	165.5 ± 8.4	0.241
Weight (kg)	81.4 ± 12.6	82.2 ± 12.7	0.754
BMI (kg/m^2^)	30.2 ± 2.9	29.8 ± 2.6	0.442
WC (cm)	96.1 ± 9.0	97.1 ± 9.6	0.577
HC (cm)	105.2 ± 5.2	103.2 ± 6.3	0.090
VFA (cm^2^)	101.9 ± 38.8	110.4 ± 34.2	0.253
SFA (cm^2^)	264.9 ± 59.5	257.3 ± 67.2	0.557
SBP (mmHg)	139.5 ± 16.1	134.0 ± 16.4	0.095
DBP (mmHg)	81.7 ± 10.7	79.4 ± 11.5	0.308
HbA1c (%)	5.5 ± 0.3	5.5 ± 0.3	0.494
FPG (mmol/L)	5.5 ± 0.5	5.5 ± 0.6	0.844
2hPG (mmol/L)	8.3 ± 1.5	8.4 ± 1.4	0.729
FINS (pmol/L)	127.9 (93.8,194.7)	130.9 (102.9,202.9)	0.326
2hINS (pmol/L)	954.6 (582.6,1382.5)	1184.0 (713.5,1552.3)	0.525
FCP (nmol/L)	1.0 (0.8,1.2)	1.1 (0.9,1.3)	0.336
2hCP (nmol/L)	4.1 (3.1,5.1)	4.6 (3.4,5.7)	0.350
HOMA‐IR	5.3 (3.9,8.1)	5.4 (3.9,9.0)	0.344
ALT (U/L)	33.0 (22.0,62.0)	32.5 (18.8,56.5)	0.302
AST (U/L)	24.0 (18.0,37.0)	24.0 (18.0,35.0)	0.721
UA (μmol/L)	388.5 ± 107.0	396.1 ± 87.4	0.709
TG (mmol/L)	1.8 ± 1.0	2.1 ± 1.2	0.179
TC (mmol/L)	5.4 ± 0.7	5.7 ± 1.3	0.142
HDL‐C (mmol/L)	1.2 ± 0.2	1.2 ± 0.2	0.764
LDL‐C (mmol/L)	3.5 ± 0.5	3.5 ± 0.8	0.766

*Note:* The values shown are mean ± standard deviation, median (interquartile range 25%–75%) or percentages. *p* values were obtained using an independent *t*‐test for normally distributed variables and the Mann–Whitney *U* test for non‐normally distributed variables.

Abbreviations: 2hCP, 2‐h postprandial c‐peptide; 2hINS, 2‐h postprandial plasma insulin; 2hPG, 2‐h postprandial plasma glucose; ALT, alanine aminotransferase; AST, aspartate aminotransferase; BMI, body mass index; DBP, diastolic blood pressure; FCP, fasting c‐peptide; FINS, fasting plasma insulin; FPG, fasting plasma glucose; HbA1c, glycated hemoglobin A1c; HC, hip circumference; HDL‐C, high‐density lipoprotein cholesterol; HOMA‐IR, homeostasis model assessment for insulin resistance; LDL‐C, low‐density lipoprotein cholesterol; SBP, systolic blood pressure; SFA, subcutaneous fat area; TC, total cholesterol; TG, triglyceride; UA, uric acid; VFA, visceral fat area; WC, waist circumference.

### 3.2. Safety and Tolerability

During the 12‐week intervention period, one participant reported mild diarrhea, which improved after symptomatic treatment. No serious adverse reactions were reported, and no participant discontinued the intervention because of serious safety concerns. However, this study did not include a formal assessment of MR‐related product characteristics, such as taste, palatability, satiety, or participant preference.

### 3.3. Changes in Anthropometric Indicators and Body Composition

The changes in anthropometric indicators and body composition in both groups after 12 weeks of intervention are shown in Table 2. There was a significant decrease in weight, BMI, waist circumference, hip circumference, VFA, and SFA in both groups compared with baseline (*p* < 0.001). Between‐group comparisons showed that the LSI + MR group achieved greater reductions in several parameters. Specifically, the reduction in weight was significantly greater in the LSI + MR group compared with the LSI group (−6.7 ± 4.2 kg vs. −4.4 ± 4.4 kg), corresponding to an additional mean weight loss of 2.3 kg (*p* = 0.013). Similarly, the decrease in BMI was greater in the LSI + MR group (−2.5 ± 1.4 kg/m^2^ vs. −1.6 ± 1.6 kg/m^2^), with a between‐group difference of 0.9 kg/m^2^ (*p* = 0.012). For body fat distribution, the reduction in VFA was also significantly greater in the LSI + MR group (−26.7 ± 24.3 cm^2^ vs. −14.6 ± 22.0 cm^2^), representing an additional decrease of 12.1 cm^2^ (*p* = 0.018). Likewise, the LSI + MR group demonstrated greater percentage reductions in weight, BMI, and VFA compared with the LSI group, with between‐group differences of 3.0%, 3.1%, and 10.8%, respectively (all *p* < 0.05).

**TABLE 2:** Comparison of the changes in anthropometric indicators and body composition after 12 weeks between the two groups.

	Changing values			Percentage of changes		
	LSI group (*n* = 42) *M* (SD)	LSI + MR group (*n* = 45) *M* (SD)	*p*_1_	LSI group (*n* = 42) (%)	LSI + MR group (*n* = 45) (%)	*p*_2_
Weight (kg)	−4.4 ± 4.4^∗∗∗^	−6.7 ± 4.2^∗∗∗^	0.013	−5.3% ± 5.5%	−8.3% ± 4.5%	0.006
BMI (kg/m^2^)	−1.6 ± 1.6^∗∗∗^	−2.5 ± 1.4^∗∗∗^	0.012	−5.3% ± 5.5%	−8.4% ± 4.5%	0.006
WC (cm)	−4.3 ± 6.1^∗∗∗^	−6.2 ± 5.8^∗∗∗^	0.145	−4.5% ± 6.4%	−6.2% ± 6.1%	0.199
HC (cm)	−2.4 ± 3.9^∗∗∗^	−2.9 ± 3.0^∗∗∗^	0.549	−2.3% ± 3.7%	−2.8% ± 2.9%	0.512
VFA (cm^2^)	−14.6 ± 22.0^∗∗∗^	−26.7 ± 24.3^∗∗∗^	0.018	−11.9% ± 21.0%	−22.7% ± 23.8%	0.032
SFA (cm^2^)	−37.4 ± 51.6^∗∗∗^	−44.3 ± 40.9^∗∗∗^	0.493	−13.2% ± 19.6%	−17.7% ± 14.7%	0.229

*Note:* The values shown are mean ± standard deviation. *p*_1_ represents the between‐group comparison of absolute changes; *p*_2_ represents the between‐group comparison of percentage changes.

Abbreviations: BMI, body mass index; HC, hip circumference; SFA, subcutaneous fat area; VFA, visceral fat area; WC, waist circumference.

^∗∗∗^ *p* < 0.001 versus baseline.

**FIGURE 2:**
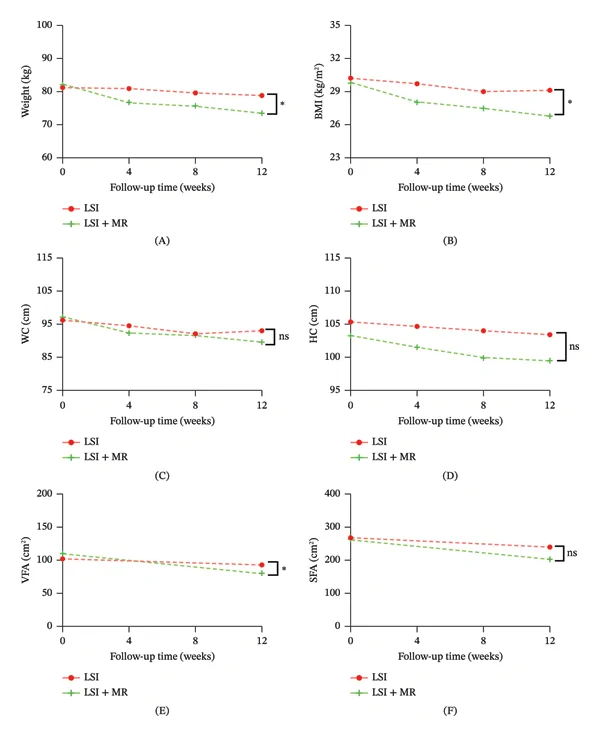
Changes in anthropometric indicators and body composition in 12 weeks between the two groups. (A) Weight, (B) BMI, body mass index, (C) WC, waist circumference, (D) HC, hip circumference, (E) VFA, visceral fat area, and (F) SFA, subcutaneous fat area. The bracket indicates the group × time interaction from the general linear model. ^∗^ *p* < 0.05; ns, not significant.

The general linear model analysis of changes in anthropometric indicators and body composition in both groups after 12 weeks of intervention is shown in Table 3. There was a significant time effect for weight (*F* = 26.692, *p* **< **0.001) and BMI (*F* = 26.345, *p* **< **0.001), implying that there was a significant decrease in weight and BMI in both groups after 12 weeks. In addition, there were significant interaction effects for weight (*F* = 5.296, *p* = 0.023) and BMI (*F* = 5.124, *p* = 0.025), indicating that the LSI + MR group showed a superior reduction in weight (82.2 ± 12.7 vs. 73.6 ± 11.0) and BMI (29.8 ± 2.6 vs. 26.8 ± 2.3) than the LSI group. Similarly, the significant time effect for VFA (*F* = 86.813, *p* **< **0.001) indicated that VFA decreased significantly in the LSI group (101.9 ± 38.8 vs. 92.9 ± 36.8) and the LSI + MR group (110.4 ± 34.2 vs. 80.2 ± 30.6) after intervention. Moreover, there was a significant interaction effect for VFA (*F* = 9.177, *p* = 0.003), signifying that the LSI + MR group had a significantly greater reduction in VFA than the LSI group. For WC, HC, and SFA, there was not a significant interaction effect for WC (*F* = 2.439, *p* = 0.120), HC (*F* = 1.328, *p* = 0.251), and SFA (*F* = 2.398, *p* = 0.123). However, there was a significant time effect for WC (*F* = 15.800, *p* **< **0.001), HC (*F* = 11.014, *p* = 0.001), and SFA (*F* = 21.636, *p* **< **0.001). After the intervention, the LSI + MR group exhibited greater reductions in WC (97.1 ± 9.6 vs. 89.5 ± 7.5), HC (103.2 ± 6.3 vs. 99.3 ± 5.2), and SFA (257.3 ± 67.2 vs. 201.1 ± 52.0) compared to the LSI group (Figure 2).

**TABLE 3:** General linear model analysis of changes in anthropometric indicators and body composition in the two groups.

Variables	Group	Baseline	12 weeks	*F* (*p* value)		
				Time	Group	Interaction
Weight (kg)	LSI (*n* = 42)	81.4 ± 12.6	79.0 ± 13.1	26.692 **(<** **0.001)**	13.235 **(<** **0.001)**	5.296 **(0.023)**
	LSI + MR (*n* = 45)	82.2 ± 12.7	73.6 ± 11.0			

BMI (kg/m^2^)	LSI (*n* = 42)	30.2 ± 2.9	29.1 ± 3.4	26.345 **(<** **0.001)**	13.977 **(0.001)**	5.124 **(0.025)**
	LSI + MR (*n* = 45)	29.8 ± 2.6	26.8 ± 2.3			

WC (cm)	LSI (*n* = 42)	96.1 ± 9.0	92.8 ± 11.2	15.800 **(<** **0.001)**	0.648 (0.422)	2.439 (0.120)
	LSI + MR (*n* = 45)	97.1 ± 9.6	89.5 ± 7.5			

HC (cm)	LSI (*n* = 42)	105.2 ± 5.2	103.3 ± 6.7	11.014 **(0.001)**	11.924 **(0.001)**	1.328 (0.251)
	LSI + MR (*n* = 45)	103.2 ± 6.3	99.3 ± 5.2			

VFA (cm^2^)	LSI (*n* = 42)	101.9 ± 38.8	92.9 ± 36.8	86.813 **(<** **0.001)**	6.452 **(0.012)**	9.177 **(0.003)**
	LSI + MR (*n* = 45)	110.4 ± 34.2	80.2 ± 30.6			

SFA (cm^2^)	LSI (*n* = 42)	264.9 ± 59.5	236.7 ± 68.0	21.636 **(<** **0.001)**	5.599 **(0.019)**	2.398 (0.123)
	LSI + MR (*n* = 45)	257.3 ± 67.2	201.1 ± 52.0			

*Note:* The values shown are mean ± standard deviation. Bold values indicate statistically significant *p* values, defined as *p* < 0.05.

Abbreviations: BMI, body mass index; HC, hip circumference; SFA, subcutaneous fat area; VFA, visceral fat area; WC, waist circumference.

### 3.4. Changes in Blood Pressure and Biochemical Indicators

The changes in blood pressure and biochemical indicators in both groups after 12 weeks of intervention are shown in Table 4. In terms of blood pressure, there was a significant time effect for SBP (*F* = 24.045, *p* **< **0.001) and DBP (*F* = 8.339, *p* = 0.004), implying that there was a significant decrease in blood pressure in both groups after the intervention, and there was a significant group effect for SBP (*F* = 7.311, *p* **= **0.008). For glycemic and insulin‐related indicators, there were significant time effects for 2hPG (*F* = 15.885, *p* < 0.001), FINS (*F* = 4.154, *p* **= **0.043), 2hINS (*F* = 7.973, *p* **= **0.005), and HOMA‐IR (*F* = 4.752, *p* **= **0.031), but no group or interaction effects. For other metabolic indicators, there were significant time effects for ALT (*F* = 18.008, *p* **< **0.001), AST (*F* = 16.359, *p* **< **0.001), TG (*F* = 9.158, *p* **= **0.003), and TC (*F* = 4.521, *p* **= **0.035), indicating that both groups showed significant improvement in liver function and decreases in TG and TC after the intervention, but no significant group or interaction effects were seen. In addition, there were no significant time effects, group effects, or interaction effects for HbA1c, FPG, UA, HDL‐C, and LDL‐C (*p* > 0.05).

**TABLE 4:** General linear model analysis of changes in blood pressure and biochemical indicators in the two groups.

Variables	Group	Baseline	12 weeks	*F* (*p* value)		
				Time	Group	Interaction
SBP (mmHg)	LSI (*n* = 42)	139.5 ± 16.1	129.1 ± 14.6	24.045 **(<** **0.001)**	7.311 **(0.008)**	0.055 (0.815)
	LSI + MR (*n* = 45)	134.0 ± 16.4	122.6 ± 12.8			

DBP (mmHg)	LSI (*n* = 42)	81.7 ± 10.7	77.4 ± 8.8	8.339 **(0.004)**	2.243 (0.136)	0.003 (0.955)
	LSI + MR (*n* = 45)	79.4 ± 11.5	75.3 ± 8.2			

HbA1c (%)	LSI (*n* = 42)	5.5 ± 0.3	5.4 ± 0.3	0.942 (0.333)	1.632 (0.203)	0.134 (0.715)
	LSI + MR (*n* = 45)	5.5 ± 0.3	5.5 ± 0.3			

FPG (mmol/L)	LSI (*n* = 42)	5.5 ± 0.5	5.3 ± 0.5	0.958 (0.329)	0.188 (0.665)	0.490 (0.485)
	LSI + MR (*n* = 45)	5.5 ± 0.6	5.4 ± 0.6			

2hPG (mmol/L)	LSI (*n* = 42)	8.3 ± 1.5	7.6 ± 1.6	15.885 **(<** **0.001)**	0.605 (0.438)	1.481 (0.225)
	LSI + MR (*n* = 45)	8.4 ± 1.4	7.2 ± 1.7			

FINS (pmol/L)	LSI (*n* = 42)	127.9 (93.8,194.7)	107.2 (70.0, 181.4)	4.154 **(0.043)**	0.271 (0.603)	1.473 (0.226)
	LSI + MR (*n* = 45)	130.9 (102.9,202.9)	84.8 (60.5,141.4)			

2hINS (pmol/L)	LSI (*n* = 42)	954.6 (582.6,1382.5)	764.4 (493.5,1032.5)	7.973 **(0.005)**	0.002 (0.964)	0.840 (0.360)
	LSI + MR (*n* = 45)	1184.0 (713.5,1552.3)	634.0 (312.6,966.4)			

HOMA‐IR	LSI (*n* = 42)	5.3 (3.9,8.1)	4.0 (2.7,7.6)	4.752 **(0.031)**	0.222 (0.638)	1.266 (0.262)
	LSI + MR (*n* = 45)	5.4 (3.9,9.0)	3.4 (2.3,5.9)			

ALT (U/L)	LSI (*n* = 42)	33.0 (22.0,62.0)	26.0 (16.0,38.5)	18.008 **(<** **0.001)**	2.486 (0.117)	0.030 (0.863)
	LSI + MR (*n* = 45)	32.5 (18.8,56.5)	19.0 (13.0,31.5)			

AST (U/L)	LSI (*n* = 42)	24.0 (18.0,37.0)	21.0 (16.8,27.0)	16.359 **(<** **0.001)**	1.088 (0.298)	0.201 (0.654)
	LSI + MR (*n* = 45)	24.0 (18.0,35.0)	19.0 (15.5,24.5)			

UA (μmol/L)	LSI (*n* = 42)	388.5 ± 107.0	377.2 ± 88.3	2.703 (0.102)	0.060 (0.806)	0.603 (0.438)
	LSI + MR (*n* = 45)	396.1 ± 87.4	363.6 ± 84.1			

TG (mmol/L)	LSI (*n* = 42)	1.8 ± 1.0	1.5 ± 0.9	9.158 **(0.003)**	0.519 (0.472)	1.447 (0.231)
	LSI + MR (*n* = 45)	2.1 ± 1.2	1.5 ± 1.1			

TC (mmol/L)	LSI (*n* = 42)	5.4 ± 0.7	5.3 ± 0.8	4.521 **(0.035)**	0.541 (0.463)	1.854 (0.175)
	LSI + MR (*n* = 45)	5.7 ± 1.3	5.2 ± 1.0			

HDL‐C (mmol/L)	LSI (*n* = 42)	1.2 ± 0.2	1.2 ± 0.3	2.927 (0.089)	0.322 (0.571)	0.029 (0.864)
	LSI + MR (*n* = 45)	1.2 ± 0.2	1.3 ± 0.2			

LDL‐C (mmol/L)	LSI (*n* = 42)	3.5 ± 0.5	3.4 ± 0.6	3.514 (0.062)	0.242 (0.623)	0.800 (0.372)
	LSI + MR (*n* = 45)	3.5 ± 0.8	3.3 ± 0.8			

*Note:* Data represent mean ± standard deviation or median (interquartile range 25%–75%). ALT, alanine aminotransferase; AST, aspartate aminotransferase; FINS, fasting plasma insulin; HbA1c, glycated hemoglobin A1c; HOMA‐IR, homeostasis model assessment for insulin resistance; TG, triglyceride; 2hINS, 2‐h postprandial plasma insulin. Bold values indicate statistically significant *p* values, defined as *p* < 0.05.

Abbreviations: 2hPG, 2‐h postprandial plasma glucose; DBP, diastolic blood pressure; FPG, fasting plasma glucose; HDL‐C, high‐density lipoprotein cholesterol; LDL‐C, low‐density lipoprotein cholesterol; SBP, systolic blood pressure; TC, total cholesterol; UA, uric acid.

### 3.5. Exploratory Subgroup Analysis According to Baseline VFA

To explore whether the effects of MR differed according to baseline visceral adiposity status, participants were stratified into two subgroups based on baseline VFA: < 100 cm^2^ and ≥ 100 cm^2^ (Table 5). Among participants with baseline VFA < 100 cm^2^, the LSI + MR group showed a significantly greater reduction in 2hPG compared with the LSI group (−1.75 ± 1.67 mmol/L vs. −0.47 ± 1.61 mmol/L, *p* = 0.023). However, no significant between‐group differences were observed in changes in weight, WC, blood pressure, HbA1c, FPG, UA, or lipid parameters in this subgroup.

**TABLE 5:** Between‐group comparisons of 12‐week changes in anthropometric and metabolic indicators stratified by baseline VFA.

	VFA < 100 cm^2^			VFA ≥ 100 cm^2^		
	LSI (*n* = 18)	LSI + MR (*n* = 20)	*p*_1_	LSI (*n* = 24)	LSI + MR (*n* = 25)	*p*_2_
ΔWeight (kg)	−4.91 ± 4.79	−6.06 ± 2.73	0.378	−4.04 ± 4.21	−7.33 ± 5.06	**0.017**
ΔWC (cm)	−4.00 ± 6.64	−4.58 ± 6.26	0.787	−4.54 ± 5.88	−7.50 ± 5.40	0.073
ΔSBP (mmHg)	−13.94 ± 15.78	−7.68 ± 13.38	0.203	−9.17 ± 17.93	−11.56 ± 15.61	0.621
ΔDBP (mmHg)	−3.67 ± 10.60	−1.68 ± 6.37	0.499	−5.67 ± 12.05	−5.68 ± 9.16	0.997
ΔHbA1c (%)	−0.02 ± 0.20	0.07 ± 0.17	0.127	−0.06 ± 0.16	−0.13 ± 0.26	0.267
ΔFPG (mmol/L)	−0.29 ± 0.46	−0.04 ± 0.48	0.113	−0.11 ± 0.54	−0.06 ± 0.58	0.742
Δ2hPG (mmol/L)	−0.47 ± 1.61	−1.75 ± 1.67	**0.023**	−0.75 ± 1.36	−0.89 ± 1.85	0.768
ΔUA (μmol/L)	−7.16 ± 65.16	−20.96 ± 75.98	0.556	−32.64 ± 104.68	−32.13 ± 82.28	0.985
ΔTG (mmol/L)	−0.40 ± 1.06	−0.25 ± 0.58	0.608	−0.30 ± 0.69	−0.99 ± 1.04	**0.01**
ΔTC (mmol/L)	−0.20 ± 0.72	−0.17 ± 0.74	0.921	0.06 ± 0.55	−0.62 ± 0.98	**0.004**
ΔHDL‐C (mmol/L)	0.07 ± 0.17	0.08 ± 0.15	0.872	0.07 ± 0.16	0.07 ± 0.17	0.93
ΔLDL‐C (mmol/L)	−0.04 ± 0.60	−0.15 ± 0.50	0.567	−0.05 ± 0.38	−0.25 ± 0.76	0.252

*Note:* Data represent mean ± standard deviation. HbA1c, glycated hemoglobin A1c; TG, triglyceride. Bold values indicate statistically significant *p* values, defined as *p* < 0.05.

Abbreviations: 2hPG, 2‐h postprandial plasma glucose; DBP, diastolic blood pressure; FPG, fasting plasma glucose; HDL‐C, high‐density lipoprotein cholesterol; LDL‐C, low‐density lipoprotein cholesterol; SBP, systolic blood pressure; TC, total cholesterol; UA, uric acid; WC, waist circumference.

Among participants with baseline VFA ≥ 100 cm^2^, the LSI + MR group achieved a significantly greater reduction in weight than the LSI group (−7.33 ± 5.06 kg vs. −4.04 ± 4.21 kg, *p* = 0.017). In addition, reductions in TG (−0.99 ± 1.04 mmol/L vs. −0.30 ± 0.69 mmol/L, *p* = 0.010) and TC (−0.62 ± 0.98 mmol/L vs. 0.06 ± 0.55 mmol/L, *p* = 0.004) were significantly greater in the LSI + MR group. No significant between‐group differences were observed in glycemic parameters, blood pressure, UA, HDL‐C or LDL‐C in this subgroup.

## 4. Discussion

This study showed that both lifestyle intervention alone and lifestyle intervention combined with MR led to significant reductions in weight and VFA after 12 weeks. Importantly, the LSI + MR group achieved greater reductions in weight, BMI, and VFA than the LSI group, suggesting that adding MR to a structured lifestyle intervention may provide additional benefit for short‐term weight and visceral fat reduction. Our research team has extensive experience in weight management [17] and provided lifestyle intervention for the control group, which also led to notable improvements in the anthropometric and biochemical indicators of the LSI group. It can be seen that lifestyle intervention is always a powerful tool in the process of weight reduction. The diet intervention in our study had a greater benefit than the previous studies that investigated MR interventions in overweight or obese subjects, which showed no significant reduction in the degree of weight between the MR group and the control group [18, 19].

The clinical relevance of our findings should be interpreted in the context of recent magnetic resonance‐based studies on prediabetes remission. In the PLIS post hoc analysis, remission of prediabetes after lifestyle‐induced weight loss was associated with greater reduction in visceral adipose tissue and improved insulin sensitivity, whereas BMI reduction was similar between responders and nonresponders [20]. This suggests that preferential reduction of visceral fat, rather than weight loss alone, may be an important mechanism underlying restoration of normal glucose regulation. A subsequent PLIS analysis further showed that prediabetes remission may occur even without weight loss and that responders were characterized by a more favorable pattern of adipose tissue redistribution, with less visceral adipose tissue accumulation, greater subcutaneous adipose tissue deposition, and improvements in insulin sensitivity, β‐cell function, and β‐cell GLP‐1 sensitivity [21]. In addition, the Tübingen Lifestyle Intervention Program demonstrated that individuals with isolated high 1‐h postload glucose had an intermediate metabolic phenotype with increased visceral adipose tissue and liver fat and were particularly responsive to lifestyle intervention, with improvements in insulin sensitivity, β‐cell function, ectopic fat deposition, and long‐term diabetes risk [22].

Together, these magnetic resonance‐based studies provide strong mechanistic evidence that prediabetes remission is closely linked to improved insulin sensitivity and favorable fat distribution, particularly involving visceral and ectopic fat. Our study is consistent with this framework and extends it in a practical intervention context. Unlike the PLIS and Tübingen studies, which used MRI and 1H‐MR spectroscopy to precisely quantify body fat distribution and hepatic fat content, our study used bioelectrical impedance analysis to estimate VFA and SFA at the umbilical level. Although this method is less precise than magnetic resonance‐based techniques, it is non‐invasive, convenient, and more feasible for routine outpatient weight‐management settings. Therefore, our findings complement the existing imaging‐based evidence by suggesting that an MR‐based dietary strategy may be a scalable approach to facilitate visceral fat reduction in individuals with prediabetes and overweight/obesity.

The magnitude of weight loss observed in the present study may also be clinically meaningful. The ADA suggests that a weight loss of 7%–10% may prevent progression from prediabetes to T2DM, based on the DPP study [23, 24]. Early studies have shown that modest weight loss of 5%–10% in patients with T2DM can improve cardiovascular‐related risk factors [25]. In our study, the LSI + MR group achieved an 8.3% reduction in body weight and a 22.7% reduction in VFA, both of which were greater than those observed in the LSI group. Previous evidence has also shown that visceral fat reduction had a greater beneficial effect on metabolic indicators than subcutaneous fat reduction [26]. Because visceral adiposity contributes to inflammation, dyslipidemia, insulin resistance, hypertension, and atherosclerosis [27, 28], weight reduction and visceral fat reduction are important therapeutic targets in individuals with prediabetes [29].

Both groups showed improvements in several metabolic indicators after the 12‐week intervention, including blood pressure, postprandial glucose, insulin‐related indices, liver enzymes, TG, and TC. However, these changes should be interpreted cautiously. In the general linear model, these variables mainly showed significant time effects, whereas most group effects and time × group interaction effects were not statistically significant. Therefore, the observed improvements in these metabolic parameters cannot be attributed specifically to the addition of MR but may reflect the effects of the shared lifestyle intervention, overall weight reduction, or increased physical activity. However, no improvement in HbA1c was observed in either group. The results were similar to those of the previous study on the implementation of MRs in overweight and obese people [30]. It is possible that it may be due to the short follow‐up period and the low mean blood glucose of the participants at baseline, which may have led to an inability to detect an effective improvement in HbA1c. Similarly, subgroup analyses within the ACOORH trial indicated that the proportion of prediabetic patients in the LSI + MR group achieving normalized blood glucose at the 12‐week follow‐up was not statistically different compared with the control group; however, statistically significant differences emerged at the 26‐ and 52‐week follow‐ups [15]. In addition, a 90‐day randomized controlled trial in an obese population showed that MR significantly reduced body weight without affecting metabolic profile [31]. The likely reason for this is that metabolic indicators were mostly normal at baseline levels.

It is notable that both groups showed significant reductions in postprandial glucose compared to baseline. However, no significant group effect or time × group interaction effect was observed for 2hPG in the overall study population. For the LSI + MR group, the reduction of postprandial glucose may be partly related to the fact that the product applied in this study was enriched with 18% whey protein [32]. The consumption of whey protein at meals has been shown to lower postprandial glucose through a variety of mechanisms, including insulin‐dependent and insulin‐independent mechanisms [33]. On the one hand, whey protein intake triggered an incretin effect and enhanced the secretion of glucose‐dependent insulinotropic peptide and glucagon‐like peptide‐1 [34]. On the other hand, the insulin‐independent effect of whey protein on blood glucose may mitigate postprandial blood glucose fluctuations by slowing gastric emptying [35, 36]. Moreover, recent research has shown that regular exercise during dietary weight loss in obese and prediabetic people can have additional metabolic benefits, such as improved insulin sensitivity [37]. Both groups of participants in our study were instructed to engage in regular exercise training, and the improvement in postprandial blood glucose may have been due in part to improved insulin sensitivity.

In the exploratory subgroup analysis stratified by baseline VFA, MR showed different patterns of benefit according to visceral adiposity status. Among participants with VFA < 100 cm^2^, LSI + MR was associated mainly with a greater reduction in 2hPG, whereas among those with VFA ≥ 100 cm^2^, LSI + MR produced greater reductions in body weight, TG, and TC. This difference may reflect the relatively lower adiposity burden in participants with VFA < 100 cm^2^, limiting the potential for further anthropometric improvement while still allowing improvement in postprandial glucose regulation. In contrast, participants with excess visceral fat may have greater obesity‐related lipid metabolic disturbances and therefore derive more evident benefits in weight and lipid reduction from energy restriction and partial dietary replacement. High‐protein MRs may also promote fat oxidation and lipid utilization during and after exercise, thereby contributing to favorable changes in body composition and lipid metabolism [38]. However, given the exploratory nature of this subgroup analysis and the limited sample size, these findings should be interpreted cautiously and confirmed in larger studies.

This study has several limitations. First, the relatively small sample size and short duration of follow‐up (12 weeks) may limit the generalizability of the findings and preclude assessment of long‐term sustainability. Second, adherence to the MR regimen was not objectively monitored using standardized methods (e.g., dietary records), which may have introduced uncertainty regarding actual intake and led to potential misclassification of compliance. Third, multiple outcomes were analyzed without formal adjustment for multiple comparisons, which increases the risk of type I error; therefore, findings for secondary endpoints should be interpreted with caution. In addition, VFA and SFA were assessed using bioelectrical impedance analysis at the umbilical level, a practical method for clinical monitoring of abdominal fat changes [39, 40]. However, because MRI and CT provide a more precise assessment of regional fat distribution, future imaging‐based studies may help further validate changes in visceral and subcutaneous fat compartments. Finally, the subgroup analysis based on baseline VFA was exploratory in nature and not prespecified, and thus the results should be interpreted as hypothesis‐generating rather than confirmatory.

Future studies should include larger, multicenter randomized controlled trials with longer follow‐up to determine whether the short‐term reductions in body weight and VFA observed with MR can be sustained and whether they translate into higher rates of prediabetes remission, reduced progression to T2DM, and improved cardiovascular outcomes. Imaging‐based methods such as MRI, CT, or 1H‐MR spectroscopy should be incorporated to validate changes in visceral, subcutaneous, hepatic, and other ectopic fat depots. Future research should also include objective measures of adherence, standardized dietary records, continuous or structured safety monitoring, and formal assessment of tolerability, satiety, quality of life, and patient‐reported outcomes. In addition, prespecified subgroup analyses are needed to determine whether individuals with higher baseline visceral adiposity, hepatic steatosis, insulin resistance, or impaired postprandial glucose regulation are more likely to benefit from MR‐based intervention. Finally, future trials should evaluate the optimal MR composition, replacement frequency, and maintenance strategy for long‐term clinical implementation.

## 5. Conclusions

Lifestyle intervention combined with MRs resulted in greater reductions in body weight and VFA than lifestyle intervention alone in individuals with prediabetes and overweight/obesity. The additional benefits of MRs appeared to vary by baseline visceral adiposity, with greater improvement in postprandial glucose among participants with lower VFA and greater reductions in body weight and lipid parameters among those with excess VFA.

## Funding

This research was funded by the Major Science and Technology Projects for Health of Zhejiang Province (Grant No. WKJ‐ZJ‐2216), Public Welfare Science and Technology Program of Ningbo (Grant Nos. 2022S182, 2024S023), and Medical Health Science and Technology Project of Zhejiang Province (Grant No. 2025KY1315).

## Disclosure

All study procedures, including randomization, interventions, and outcome assessments, were predefined in the study protocol and strictly followed throughout the study. This trial was retrospectively registered at ClinicalTrials.gov (Identifier: NCT05273840) on March 10, 2022, after the initiation of participant enrollment due to an administrative oversight. We acknowledge this as a limitation and report it here transparently, in accordance with the ICMJE guidelines for clinical trial reporting.

## Ethics Statement

Approval of the protocol was obtained from the ethics committee of The Ningbo First Hospital (Approval No. 2021‐R037, approved June 1, 2021). The study was conducted in accordance with the ethical standards in the Declaration of Helsinki. Informed consent was obtained from all subjects involved in the study.

## Conflicts of Interest

The authors declare no conflicts of interest.

## Data Availability

The data that support the findings of this study are available from the corresponding author upon reasonable request.
